# Comparative Fatigue Performance of Decarburized Surfaces in Railway Rails

**DOI:** 10.3390/ma17020290

**Published:** 2024-01-06

**Authors:** Apiwat Muttamara, Jinnaphat Sommanat, Chaosuan Kanchanomai, Ekkarut Viyanit

**Affiliations:** 1 Faculty of Engineering, Thammasat School of Engineering, Thammasat University, Khlong Luang, Pathum Thani 12120, Thailand; kchao@engr.tu.ac.th; 2 National Science and Technology Development Agency, 114 Thailand Science Park, Paholyothin Road, Klong Luang, Pathum Thani 12120, Thailand; jinnaphat.sommanat@nstda.or.th (J.S.); ekkarut.viy@nstda.or.th (E.V.)

**Keywords:** railway, rolling contact fatigue, decarburization, crack propagation, acoustic emission, residual stress

## Abstract

This study explores the comparative fatigue performance of decarburized surfaces in railway components, emphasizing rolling contact fatigue, crack propagation, and acoustic emission. The investigation entails the examination of two grades of railway steels, namely R260 and U71Mn, to analyze crack and surface characteristics subsequent to fatigue testing employing a Twin Roller Machine. The purpose is to discern the impact of decarburization on the fatigue life of these materials. The results reveal distinct patterns in crack propagation and acoustic emission between decarburized and non-decarburized surfaces, providing valuable insights into the fatigue behavior of railway components. This comparative analysis contributes to a nuanced understanding of the material’s response to cyclic loading.

## 1. Introduction

Railway components are crucial elements in transportation infrastructure, enduring intricate and dynamic loads throughout their operational life. A critical concern for their longevity and reliability is the occurrence of fatigue fractures, a phenomenon marked by initiation and growth phases [1]. The fatigue performance of railway components is crucial for ensuring the safety and reliability of railway systems. Decarburization, a process that results in the loss of carbon from the surface of steel, has been identified as a significant factor affecting the fatigue performance of railway components [1,2]. Decarburization often leads to reduced surface hardness, strength, and fatigue performance, making it a critical concern for railway applications [2,3,4].

The occurrence of decarburized surfaces on railway rails during the manufacturing process is a critical issue that affects the performance and longevity of the rails. Decarburization, which refers to the loss of carbon from the surface of the steel, can lead to reduced hardness and increased susceptibility to wear and cracking. Several factors contribute to the occurrence of decarburized surfaces on railway rails. The depth of decarburization in railway rails is influenced by the manufacturing process and the quality of the steel used [1]. Research by Deng and Deng [5] highlights that in the production of U71Mn rail, the decarburization depth is a significant concern, with the qualified percentage being lower than 60% for the requirement of less than 0.3 mm of decarburization depth. This emphasizes the importance of controlling the manufacturing process to minimize decarburization [1]. The presence of discontinuities, such as inhomogeneous microstructures and surface anomalies, can further exacerbate the detrimental effects on fatigue strength [6].

Furthermore, a study by Fosca [7] provides insight into the presence of decarburized areas on the running surface of railway rails made of carbon steel. The decarburization is attributed to strong plastic deformation due to operation and work hardening.

In addition, the work of Masoudi et al. [8] emphasizes the significance of residual stresses in inhibiting the propagation of cracks. The presence of compressive residual stress on the surface of parts is crucial for preventing crack initiation and propagation. Therefore, the control of manufacturing processes, such as quenching, is essential to ensure the presence of beneficial residual stresses and minimize decarburization. 

Many studies have also focused on the very-high-cycle fatigue (VHCF) behavior of railway wheel steel under different loading conditions, providing insights into the comparative fatigue performance of materials subjected to axial loading and rolling contact loading [9,10]. Additionally, the use of ultra-sonic fatigue testing under multiaxial loading conditions has been employed to assess the fatigue behavior of railway components, further contributing to the understanding of fatigue performance in complex loading scenarios [10]. Railway steel grade selection is a critical aspect of ensuring the safety and efficiency of railway systems. The choice of steel grade, such as R260 and U71Mn, directly impacts wear resistance, rolling contact fatigue (RCF) performance, and mechanical properties. 

Additionally, Santa et al. [11] emphasize the relationship between microstructural evolution and wear behavior of U71Mn rail steel, indicating the need for a comprehensive understanding of its properties. The mechanical behavior of rail steels under various conditions is crucial. Pan et al. [12] investigate the compressive mechanical behavior of heat-treated U71Mn rail steel over a wide range of strain rates and temperatures, providing insights into its dynamic characteristics. 

The concept of cyclic fatigue loading encompasses various phenomena such as damage, cyclic creep, and temperature increase during the loading process [13]. It involves multiaxial responses and nonproportional affine loading, as well as cross-scale processes from microscopic to macroscopic levels [14]. The approach also involves the slow propagation of cracks under cyclic loads and the determination of thermal fatigue resistance characteristics using specialized test machines [15,16]. 

Furthermore, it encompasses the estimation of fatigue strength under long-period cyclic loading and the prediction of fatigue life for load-bearing components in engineering [17]. The cyclic loading effect is considered to cancel out the stress shielding effect due to stress-induced phase transformations during the unloading process [18]. The study of RCF in railway components has been the subject of extensive research. Regazzi et al. [19] investigated the influence of deep rolling on fatigue crack growth in railway axles, highlighting the relevance of technological processes in extending the life of railway components. 

The influence of the decarburized layer on the RCF of rail materials is a critical aspect in understanding the durability and performance of railway tracks. Decarburization, which leads to the reduction of carbon content at the surface of the rail material, has been found to have a significant impact on the fatigue strength and damage mechanisms of rail steels [20,21,22]. As the depth of the decarburized layer increases, the fatigue limit of the material decreases [23]. Additionally, the decarburized layer changes the damage mechanism of the rail steel and influences the surface microstructure, hardness, wear, and fatigue resistance [24,25]. It has been observed that the reduction of the decarburized layer positively affects the material’s fatigue resistance and service performance [26,27].

Furthermore, the relationship between rail decarburization and RCF has been studied, indicating that the effect of decarburization is to increase the rail crack growth rate with increasing depth [25]. The susceptibility of railway wheels to wear and RCF damage is influenced by the properties of the wheel material, and the initiation of squats is thought to originate due to local plastic deformation of the surface from RCF loading, including dynamic wheel–rail contact forces [20,21,22].

Residual stresses, particularly in turned surfaces, have been shown to significantly influence the fatigue performance of machined components [11]. The influence of residual stresses and material properties on fatigue behavior has also been a focus of research. Sadeghi et al. [28] studied the effect of residual stresses on microstructural evolution due to RCF, highlighting the complex subsurface stress state induced by fatigue loading. These studies provide valuable insights into the multifaceted nature of fatigue behavior in railway materials. The performance of railway steel in relation to RCF and plastic deformation is a critical aspect in ensuring the safety and durability of railway infra-structure. The microstructure of railway steel, particularly pearlitic steel, has been a subject of extensive research in this context. Studies have shown that the wear performance of bainitic steel rails can have significantly better RCF performance compared to pearlitic rails [29]. 

Additionally, recent research has revealed that bainitic transformation in rail steels exhibited higher wear resistance and fatigue strengths than conventional pearlitic rail at the same hardness values [30]. Furthermore, the microstructural evolution of pearlitic wheel steels under rolling-sliding contact loading has been investigated, indicating that even in the hardest steels, some plastic deformation takes place in RCF [31]. The influence of wheel/rail contact conditions on the microstructure and hardness of railway wheels has also been explored, highlighting the impact of the properties of the wheel material on its susceptibility to wear and RCF damage [32]. Moreover, the effect of pearlite interlamellar spacing on the fatigue property of wheel steel has been studied, emphasizing the use of pearlite steel due to its high strength, hardness, and wear resistance [33].

In addition to the microstructural aspects, the plastic deformation and fatigue behavior of railway steels have been investigated. A comparative study of the cyclic deformation characteristics and fatigue behavior of head-hardened pearlitic steel and Hadfield manganese steel has been presented, shedding light on the associated microstructural changes [34]. Furthermore, the plastic deformation layer was found to be thinner in rail materials with higher hardness after the rolling test, indicating better wear resistance but more significant crack propagation and severer fatigue damage [35]. Several studies have investigated the microstructural evolution and grain refinement mechanisms in Ti-10V-2Fe-3Al alloy, providing insights into the deformation-induced grain refinement and amorphization processes. These studies underscore the significance of microstructural features in determining the mechanical properties of Ti-10V-2Fe-3Al alloy [36,37,38]. 

Acoustic emission (AE) has undergone comprehensive investigation for fatigue prediction across diverse materials. The work by Wang et al. [39] delves into the frequency characteristics of AE signals in different stages of compression and fracture, highlighting the dominant frequencies occurring during plastic failure and major fracture stages. These references offer valuable information on the use of AE signals and FFT amplitude to understand plastic deformation in the context of RCF testing. Furthermore, the research by Warhadpande et al. [40] explores the effects of plasticity on subsurface initiated spalling in RCF, shedding light on the role of plastic deformation in such phenomena. Utilized AE to monitor the fatigue dissipated energy of self-compacting rubber concrete, demonstrating the applicability of AE technology in tracking the fatigue process [41]. Additionally, the study by Ekberg et al. emphasizes that surface-initiated fatigue failures stem from severe plastic deformation of the surface material, further underlining the significance of plastic deformation in RCF [42]. 

This study undertakes a comparative analysis of the fatigue performance of decarburized surfaces in rolling contact fatigue (RCF), concentrating on the R260 and U71Mn grades. The research contributes novel insights into the repercussions of decarburization on RCF, crack propagation, and acoustic emission. The investigation maintains a controlled approach, decarburization under specific conditions, and introduces crucial advancements in comprehending peeling, crack initiation, and associated phenomena. The utilization of acoustic emission signals, coupled with FFT amplitude analysis, provides intricate details into fatigue processes.

## 2. Experimental Procedure

**Figure 1:**
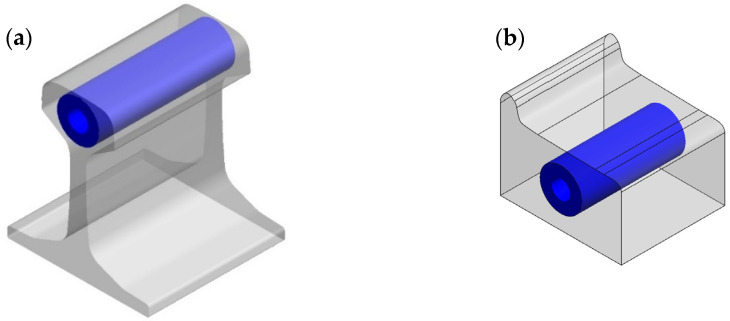
Position and Orientation of Specimens for RCF Preparation Testing (**a**) Railway Track and (**b**) Wheel.

In assessing RCF in railway components, a thorough approach was taken through material and process testing using a twin roller machine. This study broadens its focus from examining rail material to scrutinizing wheel grade, with a specific emphasis on the C class. The specimen was fabricated using wire cut EDM and subsequently ground to a diameter of 39 mm. Following this, the specimen underwent the decarburization process, involving heating the workpiece at a rate of 6 °C per minute for 4 h, followed by air cooling to room temperature. Figure 1 illustrates the position and orientation of specimens for RCF preparation testing, outlining the railway track (a) and the wheel (b). Table 1 provides the precise chemical compositions (wt.%) for two grades of railway tracks subjected to experimentation: R260 from Japan and U71Mn from China. Comparing these compositions with conventional rail steels, we find typical constituents with no extraordinary elements, indicating standardization in line with industry norms.

This comprehensive approach, designed to ensure a more complete understanding of interactions and fatigue performance within the wheel–rail system, incorporates a specific method for decarburizing the surface for comparative analysis. The testing parameters comprised applying a contact pressure of 1500 MPa between the rollers and the rail, introducing a controlled force representative of operational stresses. In order to maintain consistency while evaluating material performance under cyclic loading, the rotational speed was fixed at 200 revolutions per minute. The testing cycles were systematically increased to cover various stages, including 25,000, 50,000, 100,000, and 200,000 cycles. This incremental progression in testing provides a detailed exploration of the rail and wheel material’s fatigue characteristics and durability.

## 3. Results and Discussion

### 3.1. Materials and Processing

**Figure 2:**
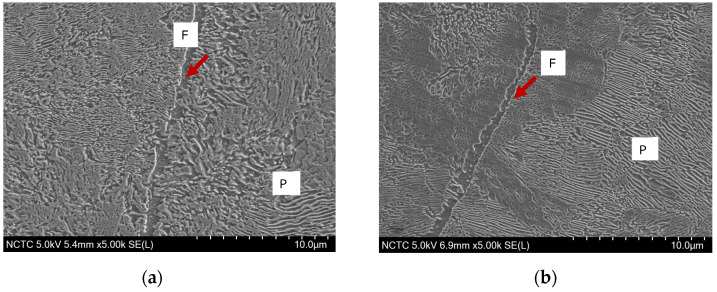
SEM micrographs of rail materials: (**a**) R260; (**b**) U71Mn.

The SEM micrographs in Figure 2 provide a detailed examination of the microstructures of two standard pearlitic rail steels, R260 and U71Mn. The images reveal almost fully the pearlitic microstructures for both R260 and U71Mn. These microstructures showcase alternating ferrite, denoted by label F in the figure, and cementite lamellae, a distinctive feature of pearlitic steels. The average spacing of pearlite lamellae, labeled P in the figure, is a critical parameter that significantly influences the mechanical properties of pearlitic steels. The observed microstructures indicate the presence of well-defined pearlite phases with discernible lamellae. The average pearlite lamellar spacing plays a vital role in determining the mechanical properties of pearlitic steels, with smaller spacings often associated with improved mechanical strength [43,44].

**Figure 3:**
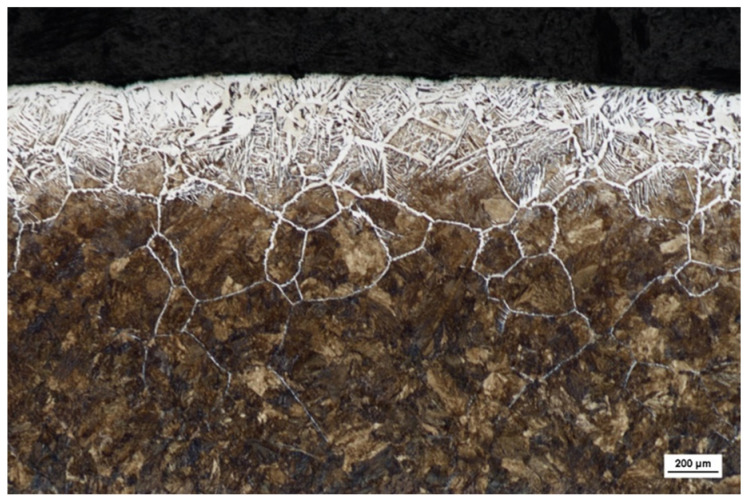
Cross-sectional Microstructure of Decarburized R260.

**Figure 4:**
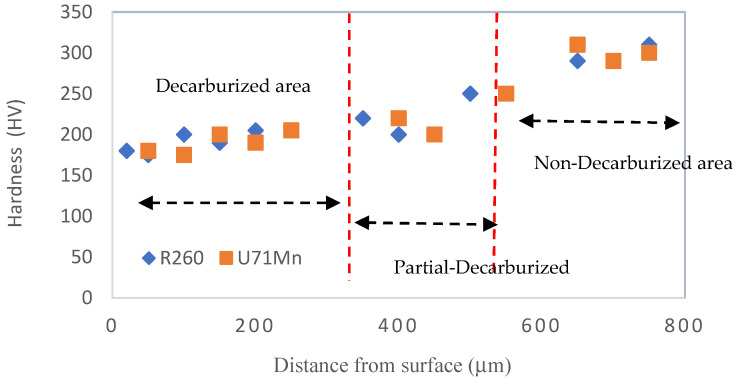
Microhardness profile from surface to depth.

Before subjecting materials to fatigue testing, a comprehensive understanding of their properties is imperative. This study focuses on the comparison of Vickers hardness in the cross-sections of two vital rail grades, R260 and U71Mn. The hardness values play a crucial role in predicting material behavior under cyclic loading. Figure 3 depicts the cross-sectional microstructure of fully decarburized specimens, and the correlation between the microstructure and hardness becomes evident. The microhardness of the cross-section profile for the two grades, R260 and U71Mn, as shown in Figure 4, was assessed using Vickers hardness with a force load of 10 g and a dwell time of 10 s. The measurements were conducted from the top surface to the specified depth along the horizontal axis, providing significant insights into the material properties. Vickers microhardness results show very similar hardness levels between R260 and U71Mn grades. 

In the fully decarburized area, the microhardness is notably lower, ranging from 180 to 200 HV. In regions of partial decarburization, there is a discernible increase in hardness compared to the fully decarburized zone, with values ranging from 190 to 260 HV. The areas with no decarburization, both decarburized and non-decarburized, exhibit higher microhardness, specifically in the range of 300 to 320 HV. This observed variation in microhardness across different decarburization states underscores the influence of surface treatment on material hardness and, consequently, its mechanical performance. The areas of full decarburization correspond to the regions exhibiting lower hardness, supporting the understanding that decarburization tends to reduce the material’s hardness [45,46]. This interplay between microstructure and hardness emphasizes the significance of decarburization in shaping the material’s mechanical characteristics.

### 3.2. Fatigue Life Cycle Assessment

**Figure 5:**
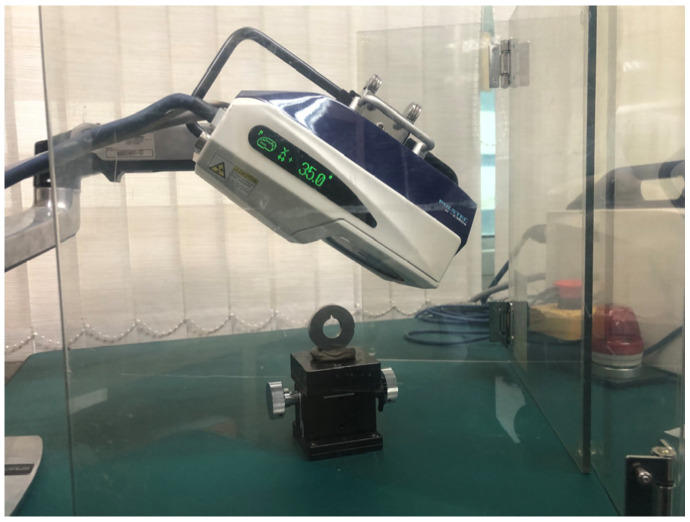
XRD Measurement Setup.

Understanding the fatigue behavior and life cycle of materials under cyclic loading is paramount, requiring a comprehensive investigation into residual stress. Residual stress plays a pivotal role in influencing the initiation and propagation of fatigue cracks, thereby shaping the overall durability and integrity of the material. Residual stress significantly influences the initiation and propagation of fatigue cracks, crucially impacting the material’s durability and integrity [47,48,49]. In this study, the μ-X360s Portable X-ray Residual Stress Analyzer by Pulstec was used to investigate residual stress. The specimen was measured four times with a rotation angle of 90 degrees, as shown in Figure 5.

**Figure 6:**
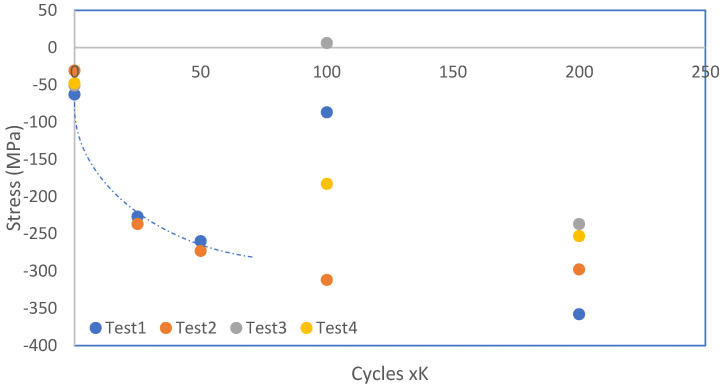
Fatigue Behavior of Decarburized Part over Cyclic Loading Cycles.

Figure 6 illustrates the fatigue behavior of the decarburized part across cyclic loading cycles. By systematically evaluating residual stress at various cycles, this research endeavors to validate that the estimated fatigue life aligns appropriately with the material’s actual service life. The presented data on residual stress for the decarburized part at different cyclic intervals (cycles K) not only contribute vital insights into the material’s behavior but also enhance our understanding of its response under cyclic loading conditions.

As the cyclic loading progresses, a discernible trend in the residual stress levels is observed. At the initial cycles (0 to 25 K cycles), the residual stress fluctuates but stabilizes around an average of −48.25 MPa. This initial phase represents the early stages of cyclic loading, and the material undergoes adjustments in its internal stress distribution. As the number of cycles increases to 50 (25 to 50 K cycles), there is a notable increase in the magnitude of residual stress, reaching an average of −266.50 MPa. This phase signifies the material’s response to prolonged cyclic loading, where the internal stress conditions continue to evolve. Further into the cyclic loading regime (100 to 200 cycles K), a complex pattern emerges. 

Residual stress values fluctuate, and there are instances of stress reversal, as evidenced by the positive value at 100 cycles K. This behavior could be attributed to the accumulation of fatigue damage and the initiation of crack propagation, leading to stress redistribution within the material. The observed variations in residual stress have direct implications for the fatigue life of the decarburized part. In fatigue testing, the cyclic loading induces stress variations that contribute to material fatigue and, eventually, failure. The initial reduction in the magnitude of residual stress indicates a stress relief phenomenon occurring in the material during the early cycles. The results consistently employ a 25 cycles K parameter across all experiments to ensure that peeling out does not occur and that the fatigue life limit is not exceeded.

### 3.3. Decarburized and Non-Decarburized for 2 Grade Railways

**Figure 7:**
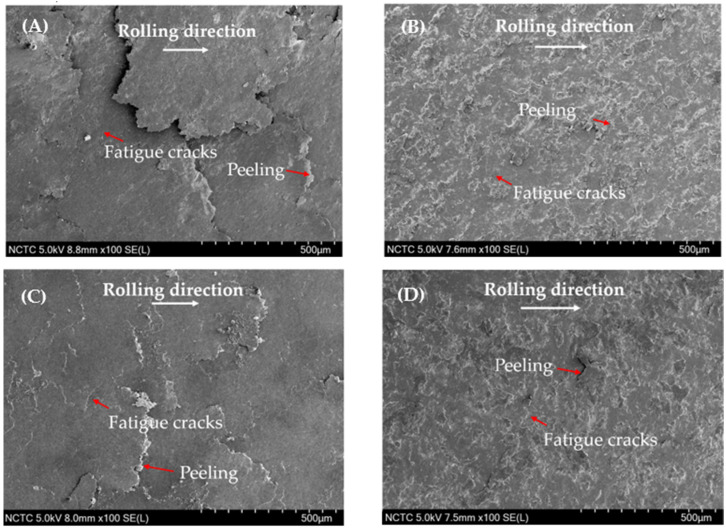
SEM micrographs of rail materials: (**A**) Decarburized-R260; (**B**) Non-Decarburized-R260; (**C**) Decarburized-U71Mn; (**D**) Non-Decarburized-U71Mn.

The comparative examination of railway components, distinguishing between decarburized and non-decarburized surfaces and emphasizing the specific grades of R260 and U71Mn, yields intriguing insights, particularly concerning phenomena such as peeling and crack formation. As illustrated in Figure 7A Decarburized-R260; Figure 7B Non-Decarburizedized-R260; Figure 7C Decarburized-U71Mn; Figure 7D Non-Decarburized-U71Mn, dedicated to decarburized and non-decarburized railway surfaces, the observations highlight distinct characteristics. Non-decarburized Figure 7B and Figure 7D surfaces consistently exhibit superior resistance to peeling across both grades. The micrographs vividly display a more favorable surface morphology in non-decarburized specimens. 

**Figure 8:**
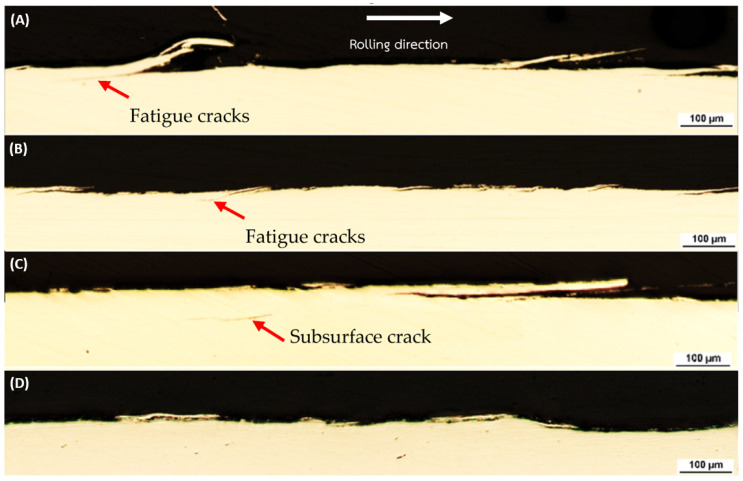
Cross-Section of Rail Materials: (**A**) Decarburized-R260; (**B**) Non-Decarburized-R260; (**C**) Decarburized-U71Mn; (**D**) Non-Decarburized-U71Mn.

This outcome aligns with established literature, emphasizing the role of pearlite in enhancing wear resistance [50,51,52,53]. Figure 8 shows cross-section of rail Materials: Figure 8A Decarburized-R260; Figure 8B Non-Decarburized-R260; Figure 8C Decarburized-U71Mn; Figure 8D Non-Decarburized-U71Mn. Crack formation, a critical factor in evaluating structural integrity, demonstrates nuanced patterns. 

Both R260 and U71Mn grades exhibit similar behavior; however, non-decarburized surfaces show superior performance over their decarburized counterparts, particularly in terms of crack initiation or propagation. Non-decarburized conditions consistently exhibit superior resistance to both peeling and crack formation, with the advantage holding true for both grades. The grade-specific variations emphasize the intricate interplay between material composition and treatment effects [54].

The enhanced fatigue resistance observed in non-decarburized surfaces suggests an improvement in the safety and reliability of railway components. The analysis of the results, as depicted in the table, provides crucial insights into the performance of decarburized and non-decarburized rail components for two distinct grades, R260 and U71Mn. In terms of peeling and crack characteristics, it is evident that non-decarburized conditions consistently showcase superior performance across both grades. The observed enhancements in peeling and crack characteristics in the non-decarburized states can be attributed to material properties. 

**Figure 9:**
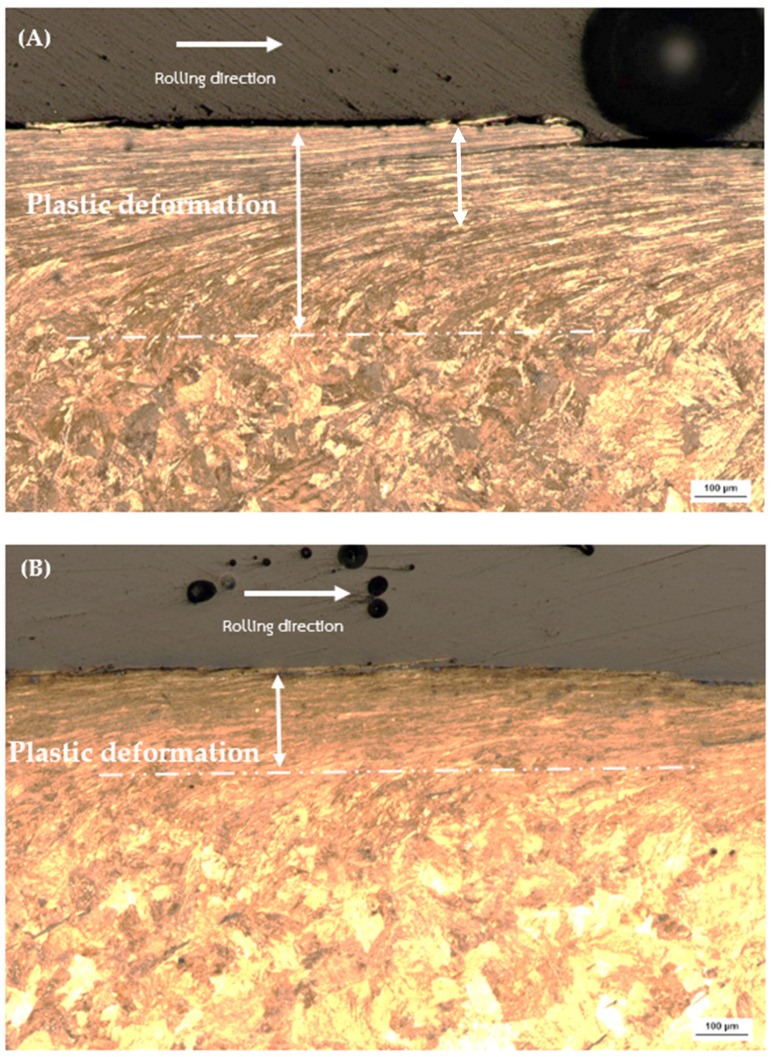
Plastic deformation of rail materials: (**A**) Decarburized R260; (**B**) Non-Decarburized R260.

**Figure 10:**
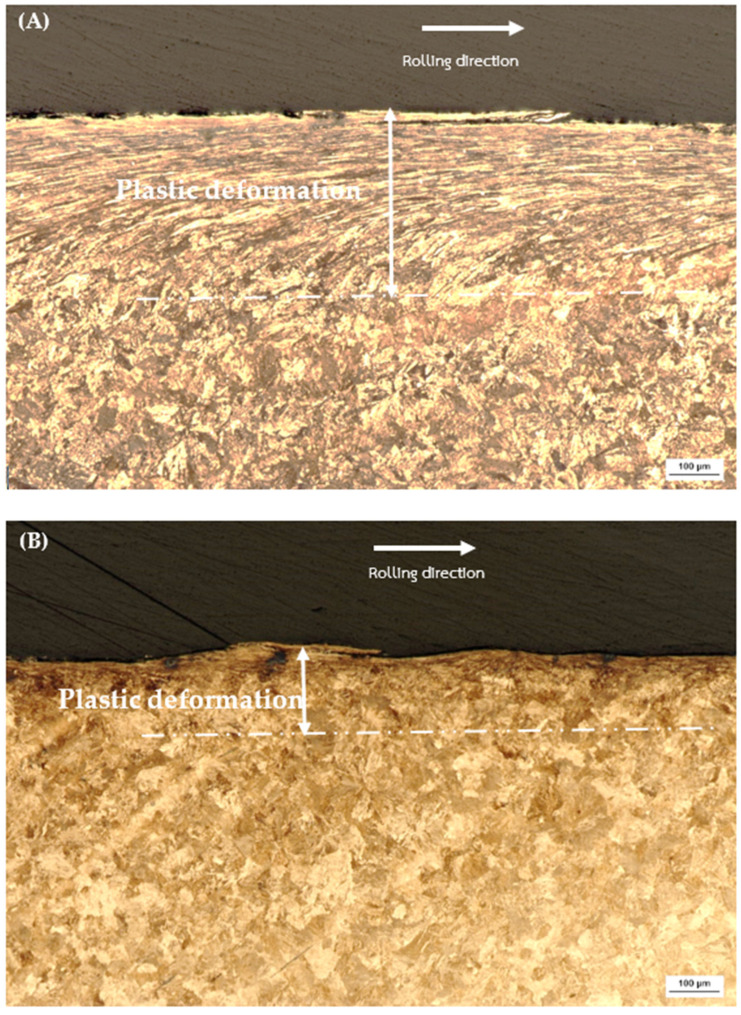
Plastic deformation of rail materials: (**A**) Decarburized U71Mn; (**B**) Non-Decarburized U71Mn.

The examination of plastic deformation in rail materials, illustrated in Figure 9 and Figure 10. Specifically, Figure 9A Decarburized R260 demonstrates a plastic deformation depth ranging from 280–310 µm, while Figure 9B Non-Decarburized R260 exhibits a shallower plastic deformation. Likewise, Figure 10A Decarburized U71Mn displays a plastic deformation depth, whereas Figure 10B Non-Decarburized U71Mn reveals a lower plastic deformation.

The noted discrepancies in plastic deformation depths provide valuable insights into the materials’ resistance to external forces. Comparing these plastic deformation depths from Figure 9, it becomes evident that non-decarburized specimens consistently experience less plastic deformation than their decarburized counterparts. This aligns with findings from the previous figure (Figure 4), where higher bulk hardness was associated with reduced plastic deformation. 

The relationship between plastic deformation depth and bulk hardness is indicative of the work-hardening characteristics of pearlitic steels. The increased plastic deformation in decarburized rail materials could be attributed to their relatively lower bulk hardness, indicating a reduced resistance to plastic deformation. On the other hand, the lower plastic deformation in non-decarburized materials, correlated with higher bulk hardness, suggests enhanced resistance to plastic deformation [51,52]. This phenomenon underscores the significance of bulk hardness in influencing the depth of plastic deformation, highlighting its role in determining the mechanical response of pearlitic steels [53,54,55].

**Figure 11:**
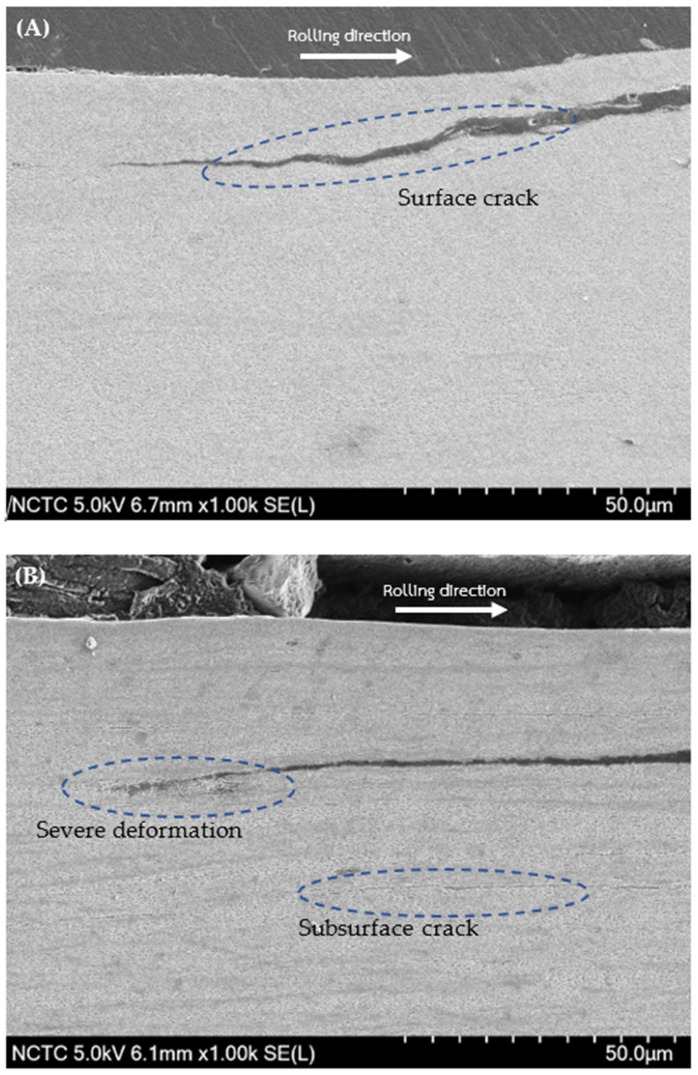
SEM micrograph of fatigue crack for R260: (**A**) Decarburized; (**B**) Non-Decarburized.

**Figure 12:**
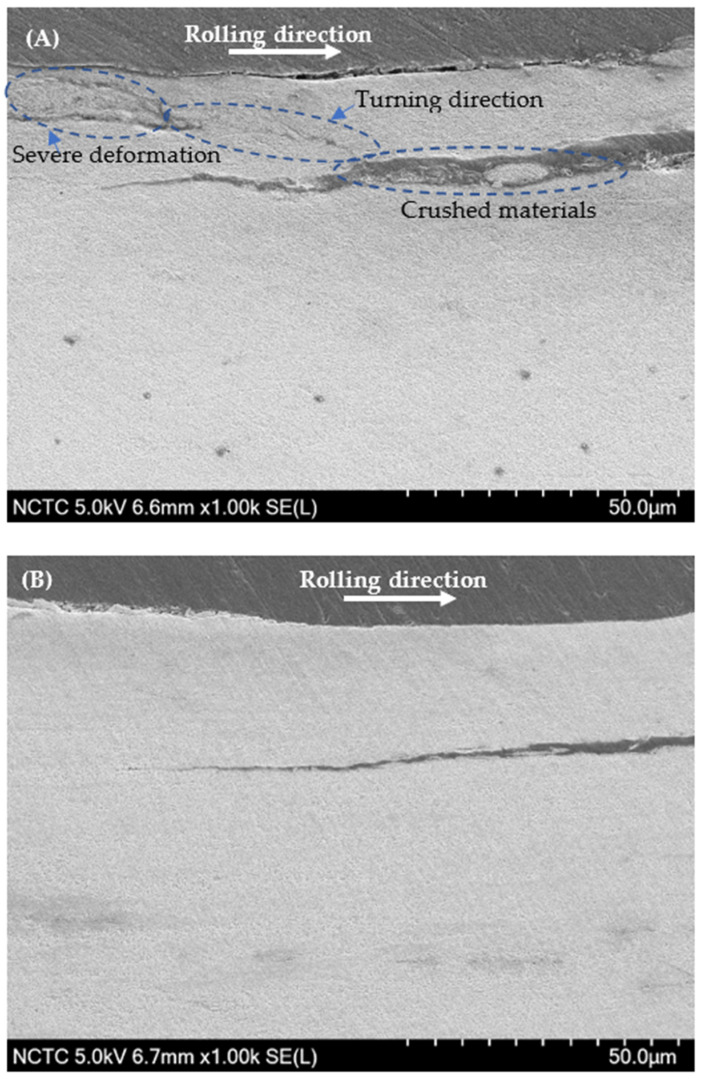
SEM micrograph of fatigue crack for U71Mn: (**A**) Decarburized; (**B**) Non-Decarburized.

In Figure 11, SEM micrographs depict fatigue cracks in R260 rail steel: Figure 11A under decarburized conditions and Figure 11B in the non-decarburized state. Similarly, Figure 12 illustrates SEM micrographs of fatigue cracks in U71Mn rail steel: Figure 12A with decarburization and Figure 12B without decarburization. The presence of decarburization in both rail grades contributes to the severity of the cracks. 

From Figure 8C, a subsurface crack can be observed. The mechanism of crack propagation from a subsurface crack caused by compressive residual stress involves; it has been observed that crack initiation and propagation occur even when the stress is significantly lower than the compressive strength of the material [56,57]. The presence of high residual stresses can lead to the initiation of subsurface cracks, particularly under cyclic compressive stress [58]. Moreover, the formation and propagation of subsurface cracks initiates from the region of the plastically deformed zone, eventually leading to surface damage [59]. Furthermore, the investigation of subsurface fatigue cracks under rolling contact has revealed the process of crack formation on the subsurface area, followed by propagation to the contact surface, emphasizing the dynamic nature of crack development in such loading conditions [60]. Hence, the surface crack depicted in Figure 11A may originate from the subsurface crack.

In Figure 12A, intense deformation arises to impede the propagation and merging of cracks in proximity. Severe plastic deformation induces substantial changes in the steel, affecting the initiation, spreading, and combination of cracks. The numerous cracks, along with the impact of severe plastic deformation, leads to the formation of crushed material within the crack [61]. Table 2 provides a comprehensive insight into the size statistics of fatigue cracks for two significant rail materials, R260 and U71Mn: decarburization and non-decarburization.

The analysis reveals significant implications for the fatigue performance of rail materials. A clear and significant difference in fatigue crack features is easily noticeable between surfaces with and without decarburization. This suggests that the R260 material, when not subjected to decarburization, maintains relatively moderate crack dimensions and angles. However, a distinct contrast is observed in the U71Mn grade under decarburized conditions, where there is a significant increase in the maximum crack length, reaching 710.39 μm. Additionally, the average length increases to 344.82 μm, and the maximum angle expands to 14.61 degrees. The relationship between crack angle and the mode of crack propagation has been investigated. It has been reported that when the crack propagation angle is less than 30°, the cracks primarily exhibit mode II behavior [62]. This indicates that the angle of crack propagation influences the mode of crack growth, which in turn affects the mechanical response of the material. Additionally, the presence of subsurface cracks, as shown in Figure 8C, has been linked to the initiation of new cracks and their propagation, emphasizing the role of crack angle in the overall damage evolution of materials [63].

This divergence in crack characteristics indicates that the decarburization process may have a more pronounced effect on fatigue crack propagation in the U71Mn grade compared to the R260 grade. Both R260 and U71Mn consistently display a material-specific response, wherein non-decarburized surfaces outshine their decarburized counterparts.

### 3.4. The Acoustic Emission (AE) Analysis

Acoustic emission (AE) is a non-destructive testing technique used to monitor and analyze the behavior of materials, including their response to plastic deformation and crack formation. The AE method involves the detection of transient elastic waves generated by the release of energy within a material as it undergoes deformation or damage.

The investigation focuses on the impact of decarburized and non-decarburized workpieces on AE. The specimen was subjected to an operating frequency ranging from 40 to 400 kHz, with a fixed resonant frequency of 150 kHz. The investigation of Acoustic Emission (AE) was conducted throughout the fatigue testing cycles, ranging from 20,000 to 20,100. The examination of these cycles, as depicted in Figure 6, reveals a continuous decrease in residual stress. The sustained reduction in residual stress implies a stable condition within this cycle range, indicative of a fatigue-resistant state in the material.

**Figure 13:**
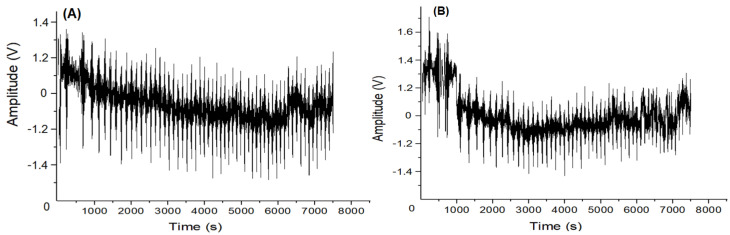
Original AE: (**A**) Non-decarburization workpiece; (**B**) Decarburization workpiece.

Figure 13 shows the original AE signal carried out on the non-decarburized workpiece (A) and the decarburized workpiece, and (B) Fast Fourier Transform (FFT) amplitude analysis in RCF testing, related to plastic deformation, underscoring the necessity of considering AE signal analysis and the frequency characteristics of FFT [64]. 

**Figure 14:**
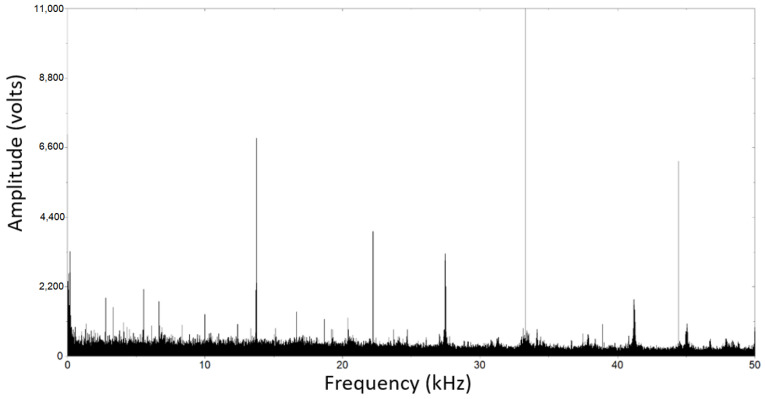
Analysis of the AE with FFT spectrum for non-decarburization workpiece.

**Figure 15:**
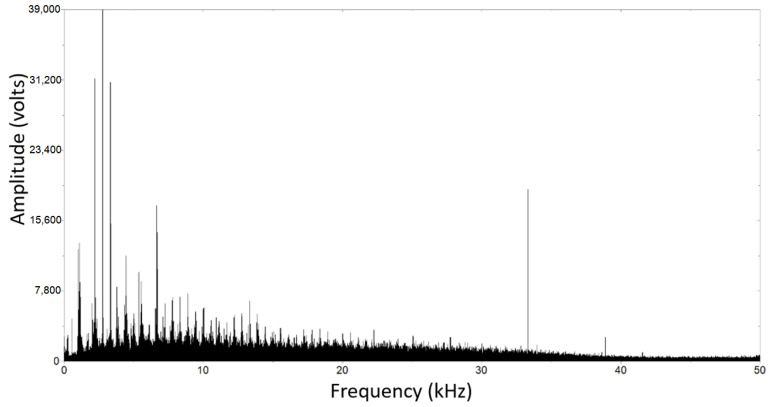
Analysis of the AE with FFT spectrum for decarburization workpiece.

The analysis of Acoustic Emission (AE) signals provides valuable insights into the behavior of materials, particularly in relation to plastic deformation and crack formation. Figure 14 illustrates the amplitude and cumulative energy of AE events related to fracture in non-decarburized workpieces, while Figure 15 portrays the AE analysis using Fast Fourier Transform (FFT) spectrum for decarburized workpieces. The observation of larger amplitudes and energy in AE signals for decarburized rail parts during fatigue life testing indicates heightened responsiveness to crack initiation and propagation. In decarburized workpieces, with higher plastic deformation depth and more cracks, there is an increased amplitude of AE signals [65]. The amplitude in AE signals is typically associated with the energy released during dynamic events like crack propagation [66,67]. Non-decarburized surfaces, with shallower plastic deformation depths and smaller cracks, exhibit comparatively lower AE amplitudes, suggesting that the severity of plastic deformation and damage is a key factor influencing AE signal amplitudes [68]. Figure 14 exhibits a lower amplitude, indicative of shallower plastic deformation depths and smaller cracks, with a maximum amplitude of 11,000 V. In contrast, Figure 15 displays a higher amplitude, associated with more severe plastic deformation depths and extensive cracks. The energy is predominantly concentrated in the range of 0 to 12 kHz, accompanied by higher frequency components exceeding 30 kHz, albeit with much smaller amplitudes, and the maximum amplitude for this figure is 39,000 V.

## 4. Conclusions

1. In the comprehensive assessment of plastic deformation and crack characteristics on railway materials, with a specific emphasis on the equivalent standards of R260 and U71Mn grades, it is evident that both grades exhibit pronounced plastic deformation and an increased occurrence of cracks, particularly on decarburized surfaces.
2. The mechanism of crack propagation from a subsurface crack, influenced by compressive residual stress, involves the interplay of various factors such as stress levels, crack initiation, propagation, and the impact of cyclic compressive stress, ultimately affecting fatigue life.
3. The utilization of Acoustic Emission (AE) signals, coupled with Fast Fourier Transform (FFT) analysis, offers a holistic insight into diverse applications. This investigative approach proves instrumental in comprehending the fracture process and detecting failure modes. It significantly contributes to the understanding of material behaviors in both decarburized and non-decarburized workpieces, each exhibiting distinct patterns of deformation and crack behavior during Rolling Contact Fatigue (RCF) testing.

## Figures and Tables

**Table 1:** Chemical Compositions (wt.%).

	C	Mn	Si	P	S	Fe
R260	0.720	0.980	0.220	0.015	0.006	Bal.
U71Mn	0.777	1.110	0.470	0.008	0.008	Bal.

**Table 2:** The size statistics of fatigue cracks for various rail materials.

Rails		Maximum Length (μm)	Average Length (μm)/S.D	Maximum Angle (Deg.)
R260	Decarburization	490.58	320.89/9	11.04
	Non decarburization	426.34	204.67/14	10.46
U71Mn	Decarburization	710.39	344.82/8	14.61
	Non decarburization	168.75	86.68/7	9.46

## Data Availability

The data presented in this study are available upon request from the corresponding author.
